# Integrated Time-Fractional Diffusion Processes for Fractional-Order Chaos-Based Image Encryption

**DOI:** 10.3390/s21206838

**Published:** 2021-10-14

**Authors:** Fudong Ge, Zufa Qin, YangQuan Chen

**Affiliations:** 1 School of Computer Science, China University of Geosciences, Wuhan 430074, China; qzf@cug.edu.cn; 2 School of Engineering (MESA-Lab), University of California, Merced, CA 95343, USA; ychen53@ucmerced.edu

**Keywords:** image encryption, spatiotemporal chaos, fractional-order Chua’s system, time-fractional diffusion processes

## Abstract

The purpose of this paper is to explore a novel image encryption algorithm that is developed by combining the fractional-order Chua’s system and the 1D time-fractional diffusion system of order α∈(0,1]. To this end, we first discuss basic properties of the fractional-order Chua’s system and the 1D time-fractional diffusion system. After these, a new spatiotemporal chaos-based cryptosystem is proposed by designing the chaotic sequence of the fractional-order Chua’s system as the initial condition and the boundary conditions of the studied time-fractional diffusion system. It is shown that the proposed image encryption algorithm can gain excellent encryption performance with the properties of larger secret key space, higher sensitivity to initial-boundary conditions, better random-like sequence and faster encryption speed. Efficiency and reliability of the given encryption algorithm are finally illustrated by a computer experiment with detailed security analysis.

## 1. Introduction

Owing to the rapid development of internet and multimedia technologies, a great number of images have been used in various applications. Since images are usually required to be confidential between the sending side and the receiver end, the protection of images by encryption becomes more and more important. During the past several decades, different techniques have been introduced to design the image encryptions [1,2]. Among them, chaos-based encryption, which was first explored by Matthews in [3], is considered as one of the most excellent encryption methods in consequence of the good property of chaos, such as high sensitivity to initial value conditions, state ergodicity and nonconvergence [4,5,6]. Based on these advantages, some more complex encryption schemes consisting of chaos and other ways, such as information entropy [7], mixed linear–nonlinear coupled map lattice [8], a pseudo-random numbers generator [9] or the genetic algorithm [10] have been investigated. However, the control parameters for permutation in above-mentioned algorithms are all fixed and the key extracted from the chaotic signals depends only on the keys, which degrade the performance of the cryptosystems.

To deal with these drawbacks, in recent years, the spatiotemporal chaos-based encryption problems have been studied, due to the fact that they can pose many excellent properties, such as a large parameter space and a random-like sequence, thereby increasing the algorithm complexity and enhancing the security. For example, in [11], a spatiotemporal chaotic cryptosystem was developed by utilizing the impulsive synchronization of reaction–diffusion systems. Based on the permutation–diffusion architecture, more sensitive chaotic image encryption schemes were proposed in [12,13]. Considering that spatiotemporal chaos is often created by local nonlinearity dynamics and spatial diffusion governed by coupled map lattices (CML), the chaos-based multiple image encryption algorithms by using CML were developed in [14,15]. For more spatiotemporal chaos-based cryptosystems, we refer the reader to [16,17] via the cellular automata, to [18] by using the generalized heat equation associated with GVTSG, or to [19] based on the DNA operation.

It is worth noting that the last decade has witnessed a significant development in the study of fractional-order chaos-based image encryption strategies [20,21,22]. Due to the introduction of the fractional-order derivatives, fractional-order chaotic systems provide additional degrees of the freedom in optimization performance, hence exhibiting more complex characteristics and having a unique advantage in the secret key space extension [23]. These make the corresponding image encryption scheme more efficient, secure and reliable.

Further, nowadays, studies indicate that the canonical diffusion systems may be inadequate to describe those anomalous subdiffusion processes observed in a spatially inhomogeneous environment, such as reheating processes of the heterogeneous metal slabs [24,25] or the spread of contaminants in underground water [26]. To improve the modeling precision of these extremely complex transport processes, the authors in [27,28,29,30,31,32] have proven that time-fractional diffusion system with a time-fractional derivative of order α∈(0,1] can be regarded as a powerful alternative model. Most importantly, we see that the time-fractional diffusion system can recover the traditional diffusion system if the order $\alpha \to 1^{-}$ [33,34]. Therefore, studies on proposing a novel spatiotemporal image encryption algorithm by integrating time-fractional diffusion system into the fractional-order chaos based image encryption should be both challenging and necessary.

Motivated by the above consideration, this paper aims to come up with a novel image encryption algorithm that is proposed by combining the fractional-order Chua’s system and the time-fractional diffusion systems of order α∈(0,1]. The reasons why we take the fractional-order Chua’s system into consideration are that (1) it is a well-known chaotic system, which generates three chaotic sequences; (2) the fractional-order Chua’s system is more simply than fractional-order Lorenz system because it requires only one nonlinear function of one variable, whereas the fractional-order Lorenz system requires two nonlinear functions of two variables; (3) most standard routes to chaos from the fractional-order Lorenz equation can be produced by the fractional-order Chua’s systems; (4) there exist some theoretical results for Chua’s system, which are absent for the Lorenz system [35]. More precisely, we first obtain the numerical solution of the fractional-order Chua’s system. Secondly, we present a novel spatiotemporal chaotic scheme by designing the chaotic sequence of fractional-order Chua’s system as the initial condition and the boundary conditions of the time-fractional diffusion system under consideration. It is revealed that the proposed image encryption algorithm, which has the properties of larger secret key space, higher sensitivity to initial boundary conditions, better random-like sequence and faster encryption speed, can gain excellent encryption performance in cryptography. To the best of our knowledge, no result is available on the topic that proposes a image encryption algorithm by integrating the time-fractional diffusion processes for fractional-order Chua’s system, which inspires this paper.

The structure of this paper is as follows. In Section 2, we provide some basic results to be used thereafter. Section 3 is devoted to giving the detailed image encryption algorithm. For the illustration, we perform a computer experiment in Section 4. The security analysis, including the histogram analysis, information entropy, adjacent pixel correlation and differential attack analysis to illustrate our results, are finally presented in Section 5.

## 2. Preliminaries

In this section, we aim to give some preliminary results to be used thereafter.

### 2.1. Fractional-Order Chua’s System

Let us consider the three-dimensional fractional-order Chua’s chaotic system [36] of the following form:

(1) $$\left\{\begin{array}{c} {}_{0}^{C}D_{t}^{q_{1}}y_{1}\left(t\right)=a\left(y_{2}\left(t\right)-y_{1}\left(t\right)-f\left(y_{1}\right)\right),\\ {}_{0}^{C}D_{t}^{q_{2}}y_{2}\left(t\right)=y_{1}\left(t\right)-y_{2}\left(t\right)+y_{3}\left(t\right),\\ {}_{0}^{C}D_{t}^{q_{3}}y_{3}\left(t\right)=-by_{2}\left(t\right)-cy_{3}\left(t\right), \end{array}\right.$$

where $y={\left(y_{1},y_{2},y_{3}\right)}^{T}$ is the state vector, ${\left(a,b,c\right)}^{T}$ is the parameter vector,

(2) $$\begin{array}{c} \begin{array}{c} f\left(y_{1}\right)=m_{1}y_{1}\left(t\right)+0.5\left(m_{0}-m_{1}\right)\left(\right|y_{1}\left(t\right)+1|-|y_{1}\left(t\right)-1\left|\right) \end{array} \end{array}$$

is the nonlinear function and $m_{0}$, $m_{1}$ are two system parameters. Here, $q_{i}\in \left(0,1\right]$, i=1,2,3, denote the order of the Caputo fractional-order derivative, ${}_{0}^{C}D_{t}^{q_{i}}$ is the Caputo time-fractional derivative given by [33]:

(3) $$\begin{array}{c} \begin{array}{c} {}_{0}^{C}D_{t}^{q_{i}}y_{i}\left(t\right)={}_{0}I_{t}^{1-q_{i}}\frac{d}{dt}y_{i}\left(t\right),\;i=1,2,3, \end{array} \end{array}$$

and ${}_{0}I_{t}^{1-q_{i}}\varphi \left(t\right)=\frac{1}{\Gamma (1-q_{i})}\int_{0}^{t}{\left(t-\tau \right)}^{-q_{i}}\varphi \left(\tau \right)d\tau$ represents the Riemann–Liouville fractional integral.

**Figure 1:**
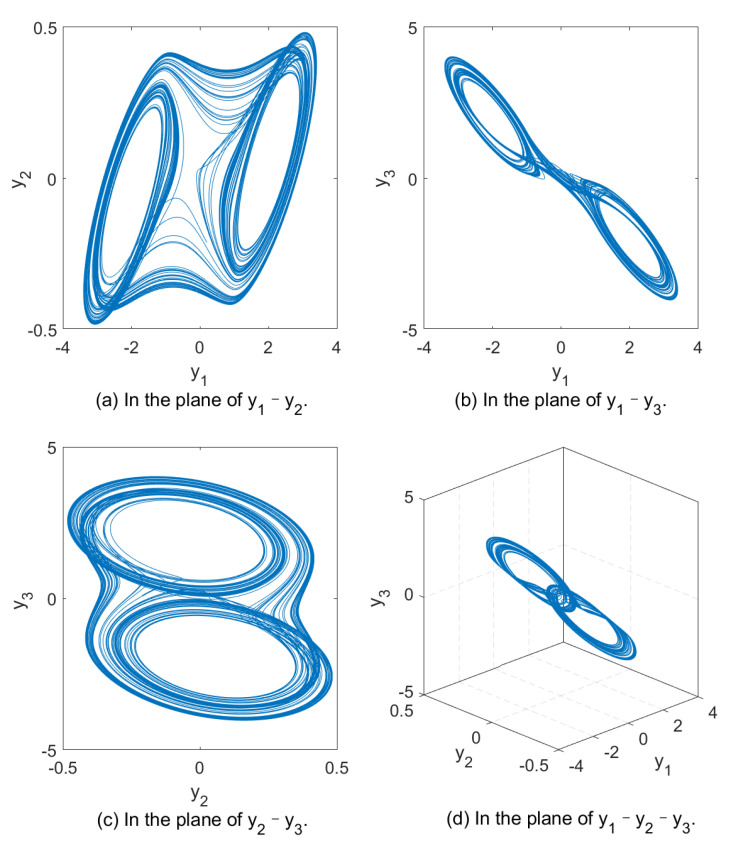
Evolution of the chaotic and its projections.

To illustrate the chaos property of above system (1), set a=10.725, b=10.593, c=0.268, $m_{0}=-1.1726$, $m_{1}=-0.7872$, $q_{1}=0.93$, $q_{2}=0.99$ and $q_{3}=0.92$, we depict the evolution of the solution for fractional-order Chua’s system (1) in Figure 1.

### 2.2. Time-Fractional Diffusion System

Throughout this paper, we consider the following time fractional diffusion equation:

(4) $$\left\{\begin{array}{c} {}_{0}^{C}D_{t}^{\alpha }\omega \left(x,t\right)=K\frac{\partial^{2}\omega \left(x,t\right)}{\partial x^{2}}+V\omega \left(x,t\right)+g\left(x,t\right),0<x<L,\;t\geq 0\\ p_{1}\omega_{x}\left(0,t\right)+r_{1}\omega \left(0,t\right)=\varphi \left(t\right),\;p_{2}\omega_{x}\left(L,t\right)+r_{2}\omega \left(L,t\right)=\psi \left(t\right),\;\;t>0,\\ \omega (x,0)=\phi (x),\;\;0<x<L, \end{array}\right.$$

where ω(x,t) denotes the system state, L>0 is a constant, *K* is the diffusion coefficient, *V* represents the reaction coefficient and $p_{1},$$p_{2},$$r_{1},$$r_{2}>0$ are four constants. Moreover, ${}_{0}^{C}D_{t}^{\alpha }$, α∈(0,1] is the Caputo time-fractional derivative with respect to *t* defined as [33]:

(5) $$\begin{array}{c} \begin{array}{c} {}_{0}^{C}D_{t}^{\alpha }y\left(\cdot ,t\right)={}_{0}I_{t}^{1-\alpha }\frac{\partial y}{\partial t}\left(\cdot ,t\right) \end{array} \end{array}$$

and φ,$\psi \in L^{2}\left[0,\infty \right)$, $\phi \in L^{2}\left(0,L\right)$ are three given functions. Here, $L^{2}\left[0,\infty \right)$ and $L^{2}\left(0,L\right)$ are, respectively, the usual Hilbert spaces with the norms induced by corresponding inner products.

**Figure 2:**
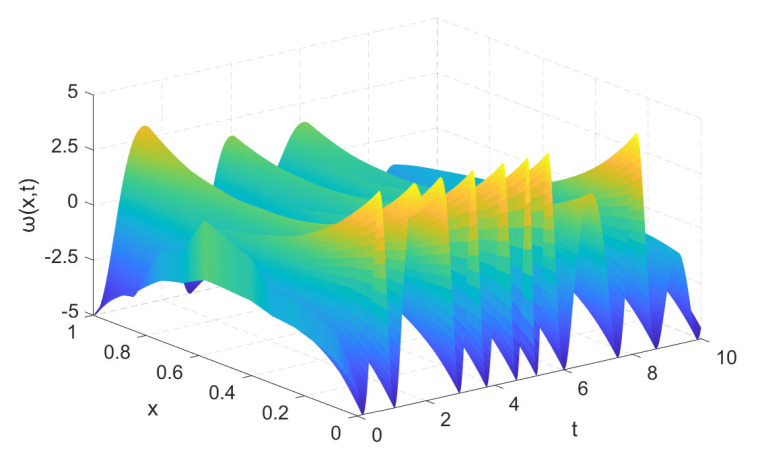
The behaviors of time-fractional diffusion system (4).

For the regularity results of this system (4), based on the eigenvalue theory of operator $▵=\frac{\partial^{2}}{\partial x^{2}}$ under the Robin boundary condition and the semigroup theory, we refer the reader to [37,38], where the detailed solution expression of time-fractional diffusion systems and its regularity results are obtained. Further, according to the high order approximation method on Caputo time-fractional derivative in [39], set

(6) $$\begin{array}{c} \begin{array}{c} \alpha =0.5,\;K=1,\;V=16,\;g\left(x,t\right)=4\sqrt{x}e^{-0.5x}\mathrm{and}p_{1}=3,\;r_{1}=1,\;p_{2}=1,\;r_{2}=2, \end{array} \end{array}$$

we extend to simulate above time-fractional diffusion system (4) and then have the Figure 2.

**Remark** **1.** *It is worth mentioning that the reason why we use the Caputo fractional-order derivative in both the fractional-order Chua’s system and time-fractional diffusion system, but not the Riemann–Liouville or the Grunwald–Letnikov formula is because of the physical significance of their initial conditions. We claim that it is okay to study the considered problems with the Riemann–Liouville or the Grunwald–Letnikov fractional-order derivatives in a mathematical sense.*

## 3. Algorithm Description

This section aims to perform a detailed description of the proposed image cryptosystem.

**Figure 3:**
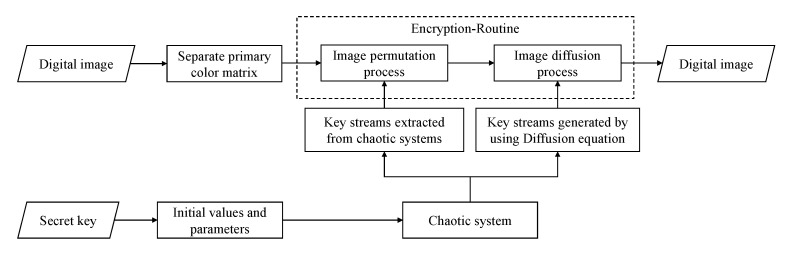
Block diagram of the proposed image encryption algorithm.

By Figure 3, the proposed spatiotemporal chaos-based cryptosystem for digital images consists of the following three parts: key stream generation, image scrambling and image diffusion.

- **Part 1. Key stream generation**: divide the three chaotic sequences generated by iterative chaotic system into nine sub-sequences;
- **Part 2. Image scrambling**: use six of the nine sub-sequences to respectively scramble the RGB primary color components of the image; design the other three sub-sequences as the initial-boundary conditions for studied time-fractional diffusion system;
- **Part 3. Image diffusion**: utilize the new sequence obtained by numerically solving the time-fractional diffusion system under consideration to diffuse the pixel values of scrambled image, thereby obtaining the encrypted image.

### 3.1. Sequence Generation and Processing

(1) Solve fractional-order Chua’s system (1) with given initial values $y_{1}\left(0\right)$, $y_{2}\left(0\right)$, $y_{3}\left(0\right)$ to obtain the three chaotic sequences. This is $\left\{y_{1}\left(i\right),y_{2}\left(i\right),y_{3}\left(i\right)\;|i=1,2,3,\ldots ,N_{1}\right\}$, where $N_{1}=N$$+4M+N_{0}$ denotes the number of iterations, $N_{0}=10,000$, and *M*, *N* are two constants determined by the size of the image.

(2) To enhance the dependence of chaotic sequence on initial values and to avoid transient effects, we discard the first $N_{0}$ values of the chaotic sequence and have a new chaotic sequence as follows:

(7) $$\begin{array}{c} \begin{array}{c} \left\{{\tilde{y}}_{1}\left(i\right),{\tilde{y}}_{2}\left(i\right),{\tilde{y}}_{3}\left(i\right)\;|i=1,2,3,\ldots ,4M+N\right\}. \end{array} \end{array}$$

After these, we separate the new chaotic sequence into nine parts, according to the rules contained in Table 1.

(3) Let the sequence of Lside, Rside and Wside be the initial boundary conditions of the time-fractional diffusion system (4). More precisely, we rewrite the initial condition and the boundary conditions of the time-fractional diffusion system (4) as follows

(8) $$\left\{\begin{array}{c} p_{1}\omega_{x}\left(0,j\right)+d_{1}\omega \left(0,i\right)=\varphi \left({\tilde{y}}_{1}\left(i\right)\right),\;\;1\leq i\leq N,\\ p_{2}\omega_{x}\left(L,j\right)+d_{2}\omega \left(L,j\right)=\psi \left({\tilde{y}}_{2}\left(j\right)\right),\;\;1\leq j\leq N,\\ \omega \left(l,0\right)=\phi ({\tilde{y}}_{3}\left(l\right)),\;\;1\leq l\leq 3M, \end{array}\right.$$

where ${\tilde{y}}_{1}\left(i\right)\in$ Lside, ${\tilde{y}}_{2}\left(j\right)\in$ Rside, ${\tilde{y}}_{3}\left(l\right)\in$ Wside.

(4) By extending the numerical method in [39], we here numerically solve the time-fractional diffusion system (4) and obtain a sequence *W* of size 3M×N. Notice that each pixel value of the image is usually an integer number ranging from 0 to 255, while the obtained sequence *W* is a real matrix. The following optimization procedure is needed:

(9) $$W_{t}=mod\left(round\left(abs\left(W-floor\left(W\right)\times 10^{m}\right)\right),256\right),$$

where abs(x) denotes the absolute value of *x*, floor(x) represents the maximum integer that is not greater than *x*, mod(x,y) means *x* mod *y* and m>0 is an integer.

(5) Splitting the optimized sequence $W_{t}$ into three matrices TX, TY and TZ with the size of M×N, one has the following:

(10) $$\begin{array}{c} \left\{\begin{array}{c} TX=W_{t}\left(1:3:end-2,:\right),\\ TY=W_{t}\left(2:3:end-1,:\right),\\ TZ=W_{t}\left(3:3:end,:\right). \end{array}\right. \end{array}$$

### 3.2. Specific Encryption Process

Suppose that *M* and *N* are, respectively, the width and height of the original digital image $I_{0}$. The detailed encryption process is given as follows.

Step 1. Separate the three primary color matrices of R, G and B of image $I_{0}$. We have the following:

(11) $$\begin{array}{c} I_{0}R=I_{0}\left(:,:,1\right),\;I_{0}G=I_{0}\left(:,:,2\right)\;\mathrm{and}\;I_{0}B=I_{0}\left(:,:,3\right). \end{array}$$

Step 2. Sort the six sequences of MX, MY, MZ, NX, NY and NZ in ascending order of numerical value respectively to obtain new array sequences. After this, we replace the value of the new array sequences with the position index of the original sequence and then, have six new array sequences: indMX, indMY, indMZ, indNX, indNY and indNZ.

Step 3. Use the above six scrambled array sequences indMX, indMY, indMZ, indNX, indNY and indNZ to scramble the rows and columns of the primary color matrix $I_{0}R$, $I_{0}G$ and $I_{0}B$, respectively, to obtain $I_{1}R$, $I_{1}G$ and $I_{1}B$. Then, we transform the matrices TX, TY and TZ and the matrices of primary color $I_{1}R$, $I_{1}G$ and $I_{1}B$ into the one-dimensional array of KX, KY and KZ and primary color component arrays of $I_{2}R$, $I_{2}G$ and $I_{2}B$ in size of MN.

Step 4. Utilize the one-dimensional arrays KX, KY and KZ to perform the ciphertext diffusion operation on the scrambled image $I_{2}$. The details are as follows:

**S4-1** Process the first pixel values of the primary color component arrays $I_{2}R$, $I_{2}G$, and $I_{2}B$ as the following:
  (12) $$\begin{array}{c} \begin{array}{cc} I_{3}R\left(1\right)= & (\left(\left(I_{2}R\left(1\right)⊕I_{2}R\left(M*N\right)\right)⊕\left(I_{2}G\left(M*N\right)⊕I_{2}B\left(M*N\right)\right)\right)⊕KX\left(1\right)),\\ I_{3}G\left(1\right)= & (\left(\left(I_{2}G\left(1\right)⊕I_{2}G\left(M*N\right)\right)⊕\left(I_{2}R\left(M*N\right)⊕I_{2}B\left(M*N\right)\right)\right)⊕KY\left(1\right)),\\ I_{3}B\left(1\right)= & (\left(\left(I_{2}B\left(1\right)⊕I_{2}B\left(M*N\right)\right)⊕\left(I_{2}R\left(M*N\right)⊕I_{2}G\left(M*N\right)\right)\right)⊕KZ\left(1\right)). \end{array} \end{array}$$
  Here, ⊕ denotes the bitwise XOR operator, and $I_{3}R$, $I_{3}G$, $I_{3}B$ are three arrays that have been diffused.
**S4-2** Encrypt the i(i≥2) element value of each primary color component array according to the formula as follows:
  (13) $$\begin{array}{c} \begin{array}{cc} I_{3}R\left(i\right)= & (\left(\left(I_{2}R\left(i\right)⊕I_{3}R\left(i-1\right)\right)⊕\left(I_{3}G\left(i-1\right)⊕I_{3}B\left(i-1\right)\right)\right)⊕KX\left(i\right)),\\ I_{3}G\left(i\right)= & (\left(\left(I_{2}G\left(i\right)⊕I_{3}G\left(i-1\right)\right)⊕\left(I_{3}R\left(i-1\right)⊕I_{3}B\left(i-1\right)\right)\right)⊕KY\left(i\right)),\\ I_{3}B\left(i\right)= & (\left(\left(I_{2}B\left(i\right)⊕I_{3}B\left(i-1\right)\right)⊕\left(I_{3}R\left(i-1\right)⊕I_{3}G\left(i-1\right)\right)\right)⊕KZ\left(i\right)). \end{array} \end{array}$$
**S4-3** Check *i*; if i≤MN, go to step **S4-2**. Otherwise, stop the loop and go to Step 5.

Step 5. A second round of ciphertext diffusion is conducted on $I_{3}R$, $I_{3}G$ and $I_{3}B$. The specific processes are:

**S5-1** Conduct the process on the primary color component arrays $I_{3}R$, $I_{3}G$ and $I_{3}B$ according to the following formula and obtain $I_{4}R$, $I_{4}G$, $I_{4}B$ following:
  (14) $$\begin{array}{c} \begin{array}{cc} I_{4}R\left(1\right)= & (\left(\left(I_{3}R\left(1\right)⊕I_{3}R\left(M*N\right)\right)⊕\left(I_{3}G\left(M*N\right)⊕I_{3}B\left(M*N\right)\right)\right)⊕KZ\left(1\right)),\\ I_{4}G\left(1\right)= & (\left(\left(I_{3}G\left(1\right)⊕I_{3}G\left(M*N\right)\right)⊕\left(I_{3}R\left(M*N\right)⊕I_{3}B\left(M*N\right)\right)\right)⊕KX\left(1\right)),\\ I_{4}B\left(1\right)= & (\left(\left(I_{3}B\left(1\right)⊕I_{3}B\left(M*N\right)\right)⊕\left(I_{3}R\left(M*N\right)⊕I_{3}G\left(M*N\right)\right)\right)⊕KY\left(1\right)). \end{array} \end{array}$$
  **S5-2** Encrypt the j(j≥2) element value of each primary color component array as follows:
  (15) $$\begin{array}{c} \begin{array}{cc} I_{4}R\left(i\right)= & (\left(\left(I_{3}R\left(i\right)⊕I_{4}R\left(i-1\right)\right)⊕\left(I_{4}G\left(i-1\right)⊕I_{4}B\left(i-1\right)\right)\right)⊕KZ\left(j\right)),\\ I_{4}G\left(i\right)= & (\left(\left(I_{3}G\left(i\right)⊕I_{4}G\left(i-1\right)\right)⊕\left(I_{4}R\left(i-1\right)⊕I_{4}B\left(i-1\right)\right)\right)⊕KX\left(j\right)),\\ I_{4}B\left(i\right)= & (\left(\left(I_{3}B\left(i\right)⊕I_{4}B\left(i-1\right)\right)⊕\left(I_{4}R\left(i-1\right)⊕I_{4}G\left(i-1\right)\right)\right)⊕KY\left(j\right)). \end{array} \end{array}$$
**S5-3** Check *j*; if j≤MN, go to process **S5-2**. Otherwise, stop the loop and then, complete the second round of ciphertext diffusion.

Step 6. Transform the three encrypted array $I_{4}R$, $I_{4}G$ and $I_{4}B$ with length MN into three matrices with size M×N, named CR, CG, CB. Finally, we use CR, CG and CB to obtain the final ciphertext image *C* by:

(16) $$\left\{\begin{array}{c} C(:,:,1)=CR(:,:),\\ C(:,:,2)=CG(:,:),\\ C(:,:,3)=CB(:,:). \end{array}\right.$$

It is worth pointing out that the encryption algorithm designed in this paper belongs to a symmetric cryptographic system and the original image can be decrypted by using the reverse encryption process.

## 4. Simulation Results

To illustrate the performance of proposed encryption strategy, we select the Lena digital image with a size of 256×256 and use the Matlab 9.4 programming platform. The parameters of the fractional-order chaotic system are a=10.725, b=10.593, c=0.268, $m_{0}=-1.1726$, $m_{1}=-0.7872$, $q_{1}=0.93$, $q_{2}=0.99$, and $q_{3}=0.92$ and the initial values read as $y_{1}\left(0\right)=0.2000$, $y_{2}\left(0\right)=-0.2120$, and $y_{3}\left(0\right)=-0.0810$. The coefficient and fractional order of the time-fractional diffusion system are:

(17) $$\begin{array}{c} \begin{array}{c} \alpha =0.5,\;K=1,\;V=16,\;g\left(x,t\right)=4\sqrt{x}e^{-0.5x}\;\mathrm{and}\;p_{1}=3,\;r_{1}=1,\;p_{2}=1,\;r_{2}=2. \end{array} \end{array}$$

**Figure 4:**
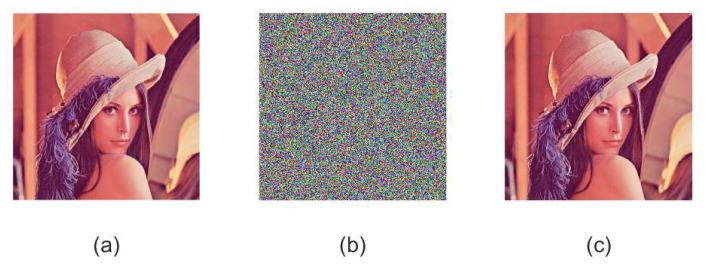
Experimental results for Lena image. (**a**) Original image of Lena, (**b**) encrypted image of Lena, (**c**) decrypted image of Lena.

Following the encryption steps presented in Section 3.2, we show the effect of using proposed image encryption algorithm to encrypt the plaintext Lena image in Figure 4. Based on this, one can find that the encrypted image is messy and is impossible to distinguish any plaintext information. Most importantly, the original plaintext image can be decrypted accurately by using the correct key. Therefore, we conclude that the proposed image encryption algorithm is secure, efficient and reliable.

## 5. The Security Analysis

In this section, several security analyses, such as key analysis, statistical analysis and information entropy analysis are performed to illustrate the quality of our proposed image encryption algorithm.

### 5.1. Key Analysis

As stated in [40], the key space of the encryption algorithm should not be smaller than $2^{100}\approx 10^{30}$ to ensure the security of the encryption algorithm. In our algorithm, the keys are divided into four parts: the initial values $y_{1}\left(0\right)$, $y_{2}\left(0\right)$, $y_{3}\left(0\right)$, the orders $q_{1}$, $q_{2}$, $q_{3}$ of the fractional-order Chua’s chaotic system, the coefficients *K*, *V* and the order α in time-fractional diffusion system. Since the accuracy of the computer is $10^{15}$ and key space of the algorithm is $10^{135}$, it is sufficient to resist exhaustive attack.

**Figure 5:**
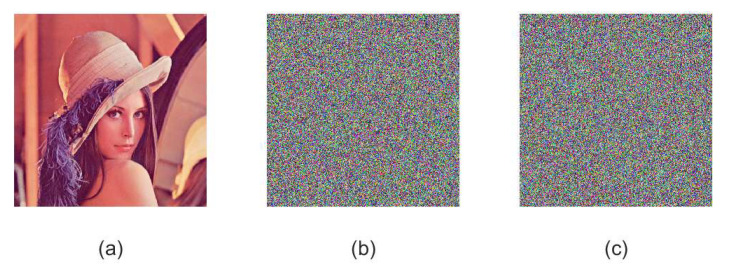
Key sensitive test. (**a**) Decrypted image with original key, (**b**) decrypted image with $y_{3}\left(0\right)=0.08100000001$, (**c**) decrypted image with α=0.50000000001.

Moreover, an efficient encryption scheme must also be sensitive to the keys. To illustrate this, suppose the two key values are changed separately: $y_{3}\left(0\right)=0.08100000001$ and α=0.50000000001. By Figure 5, it implies that our proposed algorithm has a strong key sensitivity.

### 5.2. Histogram Analysis

**Figure 6:**
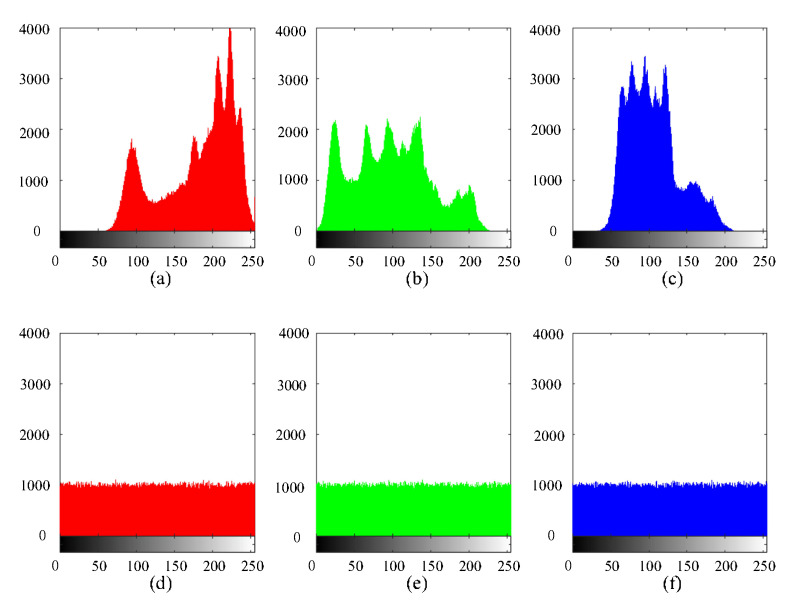
Histograms of plaintext images ((**a**) red, (**b**) green, and (**c**) blue components) and ciphertext images ((**d**) red, (**e**) green, and (**f**) blue components).

A histogram is mainly used to count the frequency of each pixel, which is an important feature of image analysis [41]. An image histogram reflects the statistical characteristics of the image, so a standard to measure the encryption effect is to make the histogram distribution of the ciphertext image as uniform as possible. To this end, we refer the reader to Figure 6, where the histogram of the plaintext image and the ciphertext image are displayed. We take into account that the pixel values of the ciphertext image on the three primary color matrices of R, G and B are more distributed than the plaintext image. Then, we obtain that the encryption algorithm proposed in this paper has a strong ability to resist statistical attacks.

### 5.3. Correlation Analysis

Notice that the correlation of adjacent pixels reflects the correlation degree of pixel values of adjacent positions in the image and a good image encryption algorithm can make adjacent pixels reach zero correlation as far as possible. More precisely, we see that the correlation of adjacent pixel points can be computed with the following formula:

(18) $$r_{xy}=\frac{cov(x,y)}{\sqrt{D(x)D(y)}},$$

(19) $$cov\left(x,y\right)=\frac{1}{N}\sum_{i=1}^{N}\left(x_{i}-E\left(x\right)\right)\left(y_{i}-E\left(y\right)\right),$$

(20) $$D\left(x\right)=\frac{1}{N}\sum_{i=1}^{N}{\left(x_{i}-E\left(x\right)\right)}^{2},$$

(21) $$E\left(x\right)=\frac{1}{N}\sum_{i=1}^{N}x_{i},$$

where $r_{xy}$ is the correlation coefficient, *x* and *y* represent pixel values, respectively, cov(x,y) is the covariance of *x* and *y*, E(x) is the mean value of *x*, D(x) is the variance of *x*, and *N* is the total number of pixels selected from the image.

**Figure 7:**
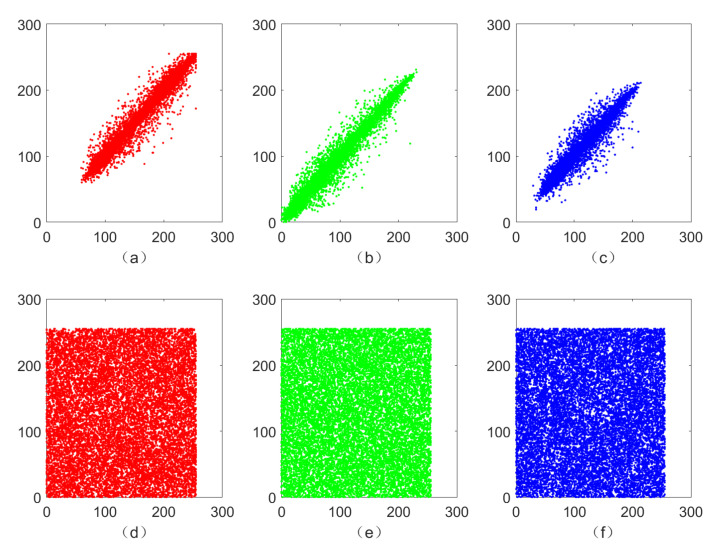
Correlation of adjacent pixels in vertical direction in plaintext images ((**a**) red, (**b**) green, and (**c**) blue components) and ciphertext images ((**d**) red, (**e**) green, and (**f**) blue components).

To this end, we randomly select 15,000 pairs of adjacent pixels from original images and encrypted image to test horizontal, vertical, and diagonal correlations and obtain Figure 7, which shows that high correlation between adjacent pixels of a plaintext image no longer exists in a ciphertext image. For more detailed values of correlation between the three color components of a plaintext image and ciphertext image in horizontal, vertical and diagonal directions, we refer the reader to Table 2. In addition, we perform a detailed comparison of correlation coefficients in horizontal, vertical and diagonal direction for several different image encryption algorithms in Table 3, which yields that the image encryption algorithm designed in this paper is more efficient and reliable.

### 5.4. Information Entropy Analysis

Image information entropy is an important indicator to measure the randomness of information in information theory. The distribution of image pixel values can be measured by information entropy as follows:

(22) $$H\left(s\right)=-\sum_{i=1}^{2^{N}-1}p\left(s_{i}\right)\log_{2}p\left(s_{i}\right),$$

where *s* denotes the information source, *N* represents the $s_{i}\left(s_{i}\in s\right)$ bit number of the symbol, and $p(s_{i})$ is the probability that the symbol $s_{i}$ appears. For a 256-level grayscale image, each pixel has $2^{8}$ possible values, and the ideal information entropy is 8. In fact, the information source difficulty generates completely random information. Therefore, the entropy of information is usually lower than the ideal value and a good cryptographic system’s information entropy should be as close to the ideal value as possible.

According to Formula (22), we obtain that the information entropy values of the ciphertext image on the three color matrices of R, G and B are 7.9993, 7.9993 and 7.9992, which are very close to the ideal expectations 8. Moreover, we refer the reader to Table 4 for more comparisons of information entropy values between the proposed algorithm and other algorithms.

### 5.5. Differential Attack Analysis

In general, the number of pixels change rate (NPCR) or the unified average changing intensity (UACI) are used to measure the encryption algorithm’s ability to resist differential attacks. So, if a slight change is made to the plaintext pixel value, a large change in the encrypted pixel value happens. This means that the encryption scheme is good. Consider that NPCR and UACI are usually given by:

(23) $$D\left(i,j\right)=\left\{\begin{array}{c} D\left(i,j\right)=0,C_{1}\left(i,j\right)=C_{2}\left(i,j\right),\\ D\left(i,j\right)=1,C_{1}\left(i,j\right)\neq C_{2}\left(i,j\right), \end{array}\right.$$

(24) $$NPCR=\frac{1}{M\times N}\sum_{i=1}^{M}\sum_{j=1}^{N}D\left(i,j\right)\times 100\%,$$

(25) $$UACI=\frac{1}{M\times N}\sum_{i=1}^{M}\sum_{j=1}^{N}\frac{|C_{1}\left(i,j\right)-C_{2}\left(i,j\right)|}{255}\times 100\%,$$

where *M* and *N* are the number of rows and columns of the image matrix, respectively, $C_{1}\left(i,j\right)$ and $C_{2}\left(i,j\right)$ represent the pixels value of the ciphertext images at the coordinate (i,j) when only one pixel value is different between two plaintext images, respectively.

As a illustration, for digital images with 256×256 pixels, the expected values of NPCR and UACI are NPCR=99.6094% and UACI=33.4635%, respectively. For this purpose, we refer the reader to Table 5, which the average values of NPCR and UACI obtained for different algorithms to encrypt Lena plaintext images are presented. Taking into account that the NPCR and UACI encryptions based on the proposed algorithm exceed 99.96% and 36.36%, respectively, we conclude that the proposed encryption algorithm is very sensitive to small changes in the plaintext image and then, has a stronger ability to carry a differential attack.

### 5.6. Speed Performance Analysis

For an encryption algorithm, the speed of the algorithm directly affects its performance, especially in the era of the rapid development of the internet. To perform the comparisons, we validate the proposed image encryption algorithm by using Matlab 9.4. The Lena plaintext image with size 256 × 256 is tested in a personal computer with a Microsoft Windows 10 64-bit operating system, Intel Core i7-7700 CPU @3.60 GHz and 8.00 GB memory. As depicted in Table 6, it is shown that the speed of our designed encryption algorithm is faster than many available encryption algorithms.

**Remark** **2.** *According to the above security analyses for illustrating the quality of the proposed encryption algorithm, the best orders of the fractional derivative in systems (1) and (4) can be determined by optimizing the penalty function that consists of the correlation coefficient, the information entropy and the numbers of NPCR and UACI. For this purpose, however, more constraints on both the studied systems and the encryption algorithms are required. This is beyond the scope of this paper. While interesting, we consider this question in our forthcoming papers.*

## 6. Conclusions

In this paper, fractional-order Chau’s system and time-fractional diffusion system with Caputo fractional derivatives are combined to greatly improve the security, efficiency and reliability of the image encryption algorithm. Simulation results and the detailed security analysis are conducted to illustrate that the image encryption algorithm proposed in this paper can gain excellent encryption performance with the advantages of larger secret key space, higher sensitivity to initial-boundary conditions, better random-like sequence and faster encryption speed. Notice that there exist several different fractional-order systems that can drive into chaos, such as the fractional-order Lorenz system, the fractional-order van der Pol system, and these fractional-order chaotic system with Riemann–Liouville or Grunwald–Letnikov fractional derivatives. Then, investigation on proposing image encryption algorithms by combining different types of fractional-order chaotic systems with more complex nonlinear fractional partial differential equations (PDEs), such as time-fractional diffusion systems with space-time-varying coefficients, space-fractional systems or a hybrid diffusion–propagation system are also of great interest. Here, the considered fractional-order chaotic systems can also be of variable-order, distributed-order or even variable-distributed-order. Furthermore, we see that the problem of determining the optimal value of parameters and orders for fractional derivatives in the considered fractional-order chaotic system and nonlinear fractional-order PDEs, which yield the best encrypt performance, is also worth discussing.

## Figures and Tables

**Table 1:** The sub-sequences of three chaotic sequences.

Names	Uses of Sequences	Number of Sequences
Lside	The initial boundary conditions of the time-fractional diffusion system (4)	$\left\{{\tilde{y}}_{1}\left(1\right),{\tilde{y}}_{1}\left(2\right),\ldots ,{\tilde{y}}_{1}\left(N\right)\right\}$
Rside		$\left\{{\tilde{y}}_{2}\left(1\right),{\tilde{y}}_{2}\left(2\right),\ldots ,{\tilde{y}}_{2}\left(N\right)\right\}$
Wside		$\left\{{\tilde{y}}_{3}\left(1\right),{\tilde{y}}_{3}\left(2\right),\ldots ,{\tilde{y}}_{3}\left(3M\right)\right\}$
MX	Scramble the rows of the primary color matrix	$\left\{{\tilde{y}}_{1}\left(3M+1\right),{\tilde{y}}_{1}\left(3M+2\right),\ldots ,{\tilde{y}}_{1}\left(4M\right)\right\}$
MY		$\left\{{\tilde{y}}_{2}\left(3M+1\right),{\tilde{y}}_{2}\left(3M+2\right),\ldots ,{\tilde{y}}_{2}\left(4M\right)\right\}$
MZ		$\left\{{\tilde{y}}_{3}\left(3M+1\right),{\tilde{y}}_{3}\left(3M+2\right),\ldots ,{\tilde{y}}_{3}\left(4M\right)\right\}$
NX	Scramble the columns of the primary color matrix	$\left\{{\tilde{y}}_{1}\left(4M+1\right),{\tilde{y}}_{1}\left(4M+2\right),\ldots ,{\tilde{y}}_{1}\left(4M+N\right)\right\}$
NY		$\left\{{\tilde{y}}_{2}\left(4M+1\right),{\tilde{y}}_{2}\left(4M+2\right),\ldots ,{\tilde{y}}_{2}\left(4M+N\right)\right\}$
NZ		$\left\{{\tilde{y}}_{3}\left(4M+1\right),{\tilde{y}}_{3}\left(4M+2\right),\ldots ,{\tilde{y}}_{3}\left(4M+N\right)\right\}$

**Table 2:** The correlation coefficient of adjacent pixels.

Direction	Plaintext Image			Ciphertext Image		
	Red	Green	Blue	Red	Green	Blue
Horizontal	0.98770	0.98831	0.97456	−0.00076	−0.00478	0.00622
Vertical	0.97527	0.97472	0.95420	0.01125	−0.01236	0.00950
Diagonal	0.96437	0.96551	0.93511	−0.00255	0.00442	0.00172

**Table 3:** Comparison of correlation coefficients for different encryptions.

	Correlation Direction		
	Horizontal	Vertical	Diagonal
The original Lena image	0.98353	0.96806	0.95499
The proposed algorithm	0.00342	0.00279	0.00120
Ref. [42]	0.07700	−0.07236	−0.06153
Ref. [15]	−0.00273	−0.00515	−0.00902
Ref. [43]	−0.00960	−0.00680	0.01447

**Table 4:** Comparison of ciphertext image information entropy for different encryptions.

	Entropy		
	Red	Green	Blue
The proposed algorithm	7.9993	7.9993	7.9992
Ref. [14]	7.9893	7.9898	7.9894
Ref. [17]	7.9971	7.9975	7.9974
Ref. [44]	7.9892	7.9898	7.9899

**Table 5:** Comparison of differential attack analysis for different encryptions.

Algorithm	Average NPCR (%)	Average UACI (%)
The proposed algorithm	99.9648	36.3651
Ref. [9]	99.6100	33.4500
Ref. [18]	99.6174	33.4404
Ref. [45]	99.6078	33.4531
Ref. [43]	99.6133	30.3633

**Table 6:** Comparison of speed performance for different encryptions.

Algorithm	Encryption Time (Seconds)
The proposed algorithm	0.0718
Ref. [7]	0.2621
Ref. [12]	0.4170
Ref. [19]	2.2234
Ref. [46]	0.1272

## Data Availability

Not applicable.
